# Immunotherapeutic progress and application of bispecific antibody in cancer

**DOI:** 10.3389/fimmu.2022.1020003

**Published:** 2022-10-20

**Authors:** Jingyue Kang, Tonglin Sun, Yan Zhang

**Affiliations:** ^1^ Lung Cancer Center, West China Hospital, Sichuan University, Chengdu, China; ^2^ State Key Laboratory of Biotherapy and Cancer Center, West China Hospital, Sichuan University, Chengdu, China; ^3^ Division of Medical Oncology, Cancer Center, West China Hospital, Sichuan University, Chengdu, China

**Keywords:** bispecific antibody, bsAb, antibody constructs, clinical trials, tumor immunotherapy

## Abstract

Bispecific antibodies (bsAbs) are artificial antibodies with two distinct antigen-binding sites that can bind to different antigens or different epitopes on the same antigen. Based on a variety of technology platforms currently developed, bsAbs can exhibit different formats and mechanisms of action. The upgrading of antibody technology has promoted the development of bsAbs, which has been effectively used in the treatment of tumors. So far, 7 bsAbs have been approved for marketing in the world, and more than 200 bsAbs are in clinical and preclinical research stages. Here, we summarize the development process of bsAbs, application in tumor treatment and look forward to the challenges in future development.

## 1 Introduction

The development of bsAb began in 1961, Nisonoff and his colleagues linked the Fab fragments of two different rabbit antibodies by reoxidation, and proved that the product can recruit two different types of cells, and proposed the concept of bsAb (1). In 1975, Milstein and Kohler fused splenic B lymphocytes and myeloma cells from immunized mice to form hybridoma cells that could produce monoclonal antibodies (2). In 1983, Milstein et al. produced the first bsAb with an intact Immunoglobulin G (IgG) structure using two fused hybridoma cells. In 1985, Perez et al. demonstrated that bsAbs that can bind to T cell receptors and tumor-specific antigens can attract T cells to tumor sites and induce T cells to kill tumors (3, 4), enhancing antibody-dependent cell-mediated cytotoxicity (ADCC). With the continuous advancement of antibody technology, double-antibody drugs have gradually begun to appear in the market. In April 2009, the EMA approved the first therapeutic bsAb catumaxomab developed by Trion Pharma in Europe for the intraperitoneal treatment of patients with malignant ascites (5). Although catumaxomab was withdrawn from the market in 2017 due to poor sales and other reasons, research on it is still ongoing. In December 2014, the FDA approved blinatumomab developed by Amgen for the treatment of acute lymphoblastic leukemia (6), which is also the first approved drug targeting CD19. In November 2017, the FDA approved emicizumab developed by Roche for the treatment of hemophilia A (7). In May 2021, the FDA approved amivantamab, developed by Janssen, for the treatment of *EGFR*-mutated non-small cell lung cancer (8). In January 2022, the FDA approved faricimab, developed by Genentech, for the treatment of wet age-related macular degeneration (w-AMD) and diabetic macular edema (DME) (9). In June 2022, the FDA approved mosunetuzumab developed by Roche for the treatment of adult patients with relapsed/refractory follicular lymphoma (R/R FL) who have received at least two prior systemic therapies. China Medical Products Administration (NMPA) approved candonilimab developed by Akeso for the treatment of relapsed or metastatic cervical cancer (R/M CC) in June 2022. With the successful listing and effective clinical application of bsAbs, it has gradually become a hot spot in antibody drug research. Currently, there are more than 200 bsAbs in clinical and preclinical research stages. This article will summarize and discuss the structure type, mechanism of action, clinical application and future challenges of bispecific antibodies (**Table 1**).

**Table 1:** Timeline of bsAb development.

Time	Incident
1960	First mention of the bsAb concept.
1964	First demonstration of the bsAb concept. First fragment-based format.
1975	Hybridoma technology pioneered by Köhler and Milstein.
1983	Hybrid hybridoma (quadroma) pioneered. First asymmetric format.
1985	First demonstration of T cell redirection.
1988	Invention of the scFv fragment.
1993	First recombinant fragment-based formats.
1995	First solution to LC-association issue through species-restricted LC pairing.
1996	First solution to chain-association issue through use of complementary HCs (knobs into holes) and common LCs.
1997	First symmetric format.
1999	Discovery that natural human lgG4 is bispecific.
2007	Discovery and elucidation of bispecific human IgG4 Fab-arm exchange process *in vivo*. DVD-lg symmetric format pioneered.
2009	Catumaxomab approved in the EU, it was withdrawn in 2017 for commercial reasons.
2011	Domain crossover as solution to LC-association issue.
2012	Generation of bispecific IgG1 through Fab-arm exchange of separately expressed antibodies.
2014	Blinatumomab approved in the United States. First orthogonal Fab interface as solution to LC-association issue.
2017	Emicizumab approved in the United States.
2021	Amivantamab approved in the United States.
2022	Faricimab approved in the United States.
	Mosunetuzumab approved in the United States.
	Candonilimab approved in China.

## 2 Bispecific antibody

**Figure 1:**
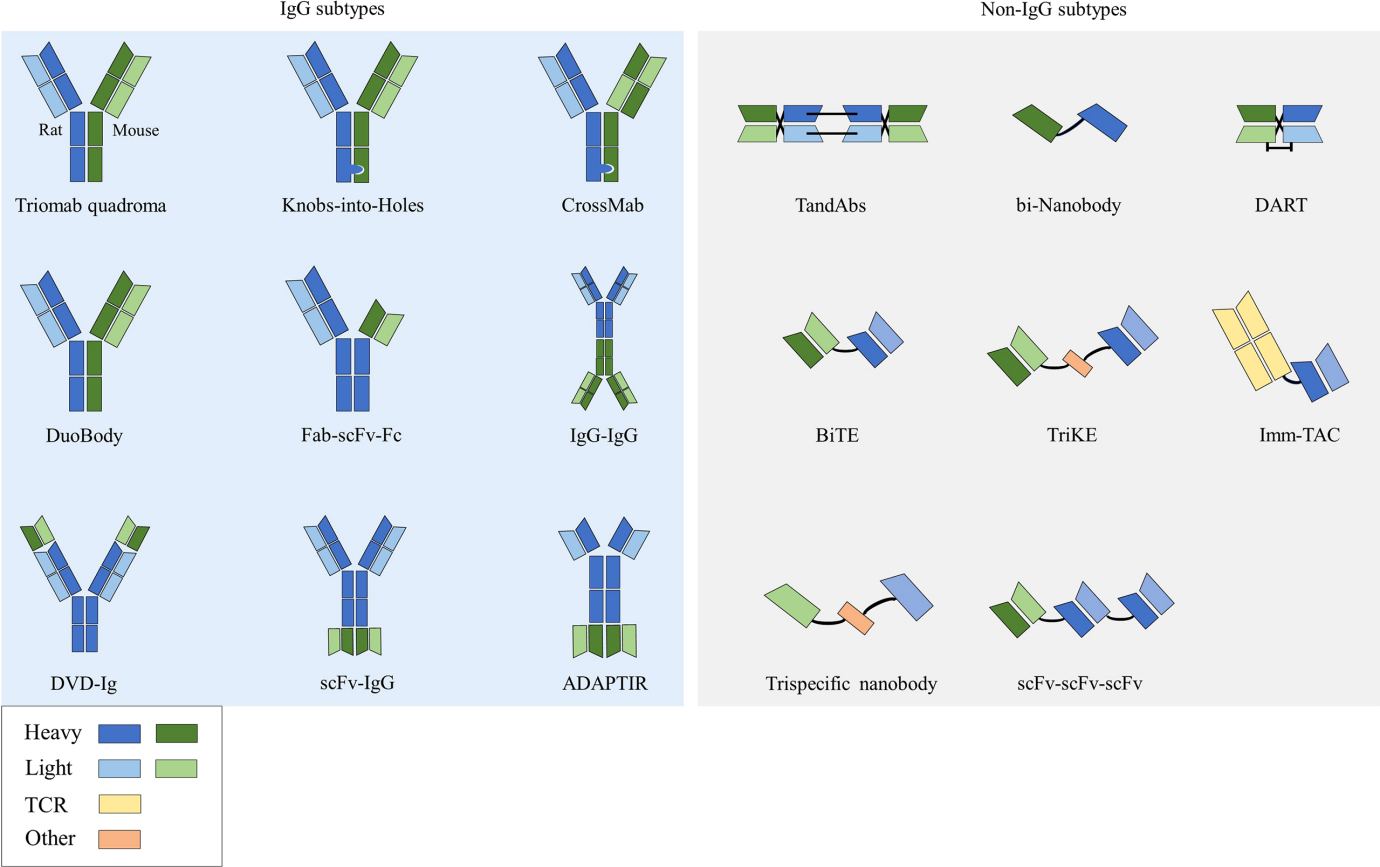
Structures of bsAbs.

Bispecific antibodies (bsAbs) have two distinct antigen-binding sites and can recognize two distinct epitopes on the same or different antigens (10). According to the structure, bsAbs can be roughly divided into two categories: IgG-like subtypes containing an Fc region and non-IgG-like subtypes without an Fc region (11) (**Figure 1**).

### 2.1 Structures of bsAbs

#### 2.1.1 IgG subtypes

This type of bsAb is based on the structure of full-length IgG. Because it has an Fc region, it has the advantages of good solubility and stability, long half-life (12), and enhanced tumor killing effect through ADCC and complement dependent cytotoxicity (CDC) effects. Among them, the symmetrical type has a structure and stability similar to that of natural IgG, with mature technology and high expression. However, its differences in structure and size from native antibodies can negatively affect related favorable properties such as stability and solubility, and thus may lead to impairments in physicochemical or pharmacokinetic properties (13). Due to the symmetry of the class, most formats in clinical development are characterized by the close proximity of antigen-binding sites, which may affect bispecific binding and even lead to reduced functional potency. The asymmetric type solves the technical bottleneck of Knobs-into-Holes in common light chain, realizes the bivalent binding of tumor antigens and the monovalent binding of CD3, and can reduce the toxicity of CD3 antibodies when binding tumor antigens. However, there are also problems such as long technical route and high difficulty in design process in its preparation process. There are many technical platforms for the preparation of IgG subtype bsAbs, among which the platforms for producing asymmetric bsAbs include Triomab quadroma, Knobs-into-Holes (KiH), CrossMab, DuoBody and so on. Platforms for producing symmetrical bsAbs include DVD-Ig, FynomAb, Two-in-one/DAF, etc. Only TrioMab and CrossMab are clinically validated. However, since catumaxomab has been withdrawn from the market, strictly speaking, only CrossMab has obtained clinical validation. Other platform products are in the early and mid-stage clinical trials.

##### 2.1.1.1 Triomab quadroma

**Figure 2:**
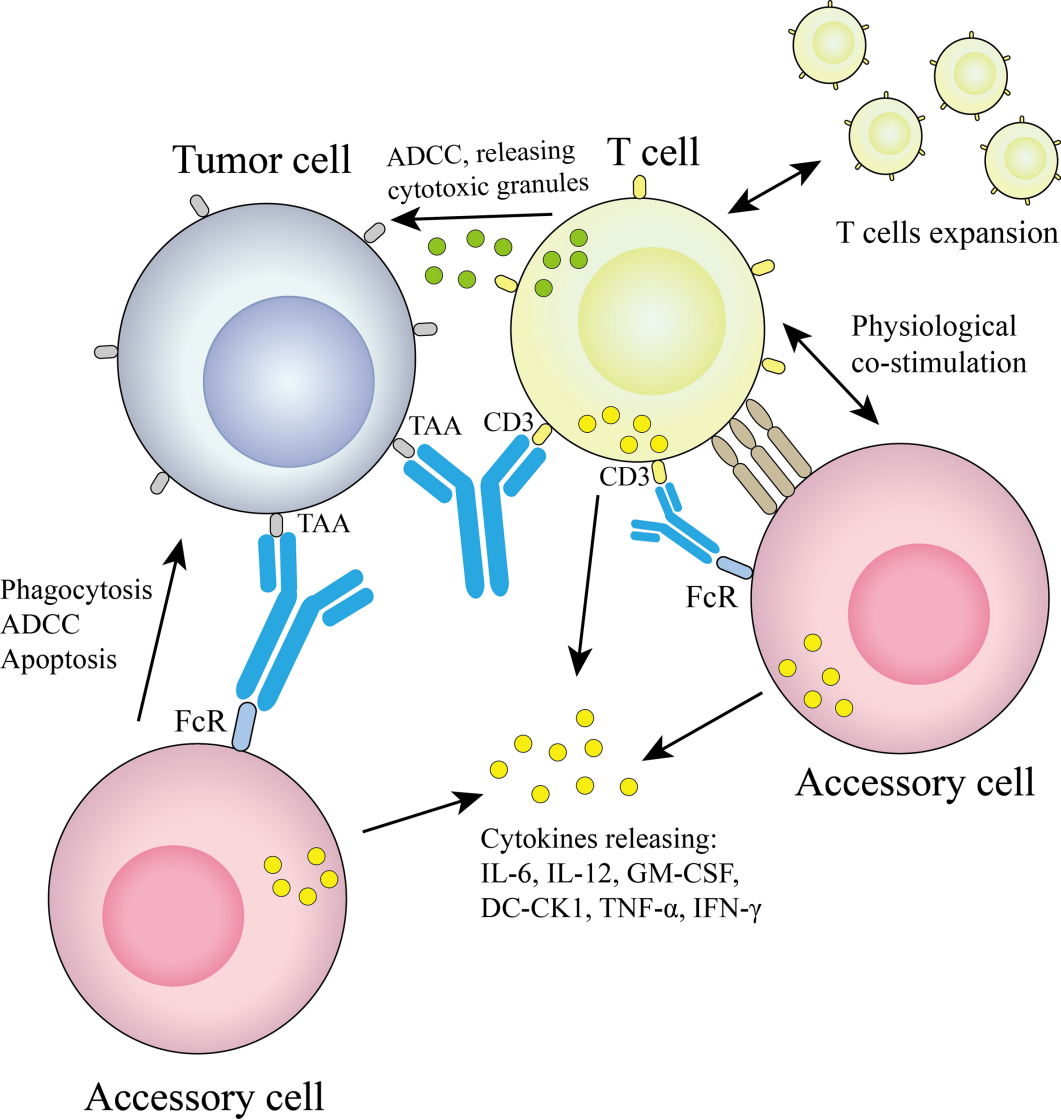
Triomab antibodies redirect T cells and other accessory cells to a tumor cell.

The technology platform, jointly developed by Fresenius and TriOn Pharma, is based on the somatic fusion of two different hybridoma cells to generate monoclonal antibodies with the desired specificity (14), which are structurally similar to conventional antibodies. Since quadroma produces antibodies by randomly assembling two different heavy chains and two different light chains, many different structures are produced, but only one of them is the desired functional bsAb. A chimeric quadroma technology was later developed by fusing murine and rat hybridoma cell lines (15), which enriches functional bsAbs and reduces mismatches. The bsAb catumaxomab targeting EpCAM and CD3 was developed based on this platform and consists of mouse IgG2a and rat IgG2b (5). The mechanism of Triomab quadroma is shown in **Figure 2**.

##### 2.1.1.2 Knobs-into-Holes

KiH was developed by Genentech, which involves engineering the CH3 domain to create a “knob” or a “hole” on each heavy chain to promote Fc heterodimerization (16) and reduce mismatches. A small molecule amino acid in the CH3 domain is replaced with a large molecule amino acid, resulting in a knob, and a large molecule amino acid in another CH3 domain is replaced with a small molecule amino acid, resulting in a hole, and the knob binds to the hole to promote heterozygous dimerization (17). The heterodimer formation mediated by this technology can provide more than 90% of the desired product under co-expression conditions, resulting in large-scale production to meet clinical and market needs (18). While KiH technology solves the problem of heavy chain mismatches, the problem of light chain mismatches remains to be solved, and random light chain binding can cause unwanted antibodies to mix with desired antibodies. In response to this problem, researchers have proposed some methods: 1) Generate bsAbs with a common light chain (19). But this method is not applicable to all bsAbs, and specific binding may be limited; 2) Knob-containing and hole-containing half-molecules are expressed in different bacteria, respectively. This approach avoids mispairing of light chains, but expression in bacterial cells also results in the loss of key glycosylation modifications, which may affect antibody effector function (20); 3) binds CrossMab and knobs-into -holes. In CrossMab antibodies, the CH1 domain of the heavy chain was exchanged with the CL domain of the corresponding light chain to induce correct pairing of the light chain (21); 4) additional mutations were introduced in the VH-VL and CH1-CL interface. These mutations prompt the heavy chain to preferentially pair with the light chain (22), but the disadvantage is that it requires extensive mutation in conserved regions of the antibody.

##### 2.1.1.3 CrossMab

CrossMabs were developed by Roche and are based on the intra-arm cross-exchange of the Fab domains of bispecific IgG antibodies, allowing for correct chain binding and correct heterodimerization of heavy chains *via* knob-into-hole or charge interaction to achieve (23–25). There are various ways of exchange, which can be Fab domains (CrossMabFab format), variable VH-VL domains in Fab fragments (CrossMabVH-VL format) and invariant CH1-CL domains (CrossMabCH1-CL format). The technology is proven and mature, becoming one of the most versatile antibody engineering techniques in industry and academia. To date, at least eight different bsAbs using CrossMab technology have entered clinical development (26), and many are in late preclinical development. The bsAb Vanucizumab developed by Roche targeting VEGF-A and angiopoietin 2 (Ang-2) is prepared by CrossMAb technology: CL and CH1 in the Fab domain of Ang-2 antibody are exchanged using CrossMAb technology, The Fab structure of VEGF antibody remains unchanged. The modified Ang-2 antibody light chain is not prone to mismatch with the heavy chain of VEGF antibody. At the same time, the “knob-into-hole” structure can promote the heterodimerization of the two heavy chains. change (27).

##### 2.1.1.4 DuoBody

The technology platform was developed by Genmab. The DuoBody technology generates bsAbs through a controlled Fab-arm exchange redox reaction of two parental homodimeric antibodies (28). Parental mAbs are expressed and pooled separately prior to selective reduction of disulfide bonds in the hinge region. Chains are recombined to a bispecific molecule, which is facilitated by the amino acid substitutions in the CH3 domain to drive preferential chain association of a heterodimer. DuoBody preserves the structural integrity of the homodimeric mAb and preserves Fc function. This process can generate four types of bsAbs: two combinations of symmetrical unoxidized, symmetrically oxidized, and asymmetrically oxidized (29). The process of producing bsAbs using the DuoBody generally involves three basic steps: 1) separate production of monospecific antibodies with the corresponding mutations in mammalian cell culture, 2) purification according to standard procedures, 3) in specific experiments Fab-arm exchange was performed under chamber conditions, followed by another purification step. This typically yields bsAbs with heterodimer content greater than 95% (30). The bsAb Amivantamab targeting mesenchymal transition factor (MET) and epidermal growth factor receptor (EGFR) was developed using this platform for the treatment of advanced non-small cell lung cancer.

##### 2.1.1.5 DVD-Ig

The technology platform was developed by Abbott, and its product is a bispecific tetravalent IgG-like molecule containing an Fc region and a constant region, and each arm of the molecule contains two variable domains (VDs): an external VD or VD1 composed of VH1 and VL1; and an internal VD or VD2 adjacent to CH1 and CL and composed of VH2 and VL2 (31). VD1 and VD2 are linked by flexible linkers with different binding specificities, and they can theoretically be synthesized by linking variable region sequences from any pair of mAbs (32). Linker is usually the sequence connecting VH-CH1 and VL-CL in IgG1 mAb, and it can also be the amino acid sequence or G4S sequence of IgG1 hinge region. This technique avoids mismatches between different light and heavy chains, but has the disadvantage that the affinity of the internal VD or VD2 may be reduced. The keys to the design of DVD-Ig molecules are (1): the selection of VD pairings from the parental mAb (2), the inner/outer orientation of the selected VDs, and (3) the amino acid sequence used to link the VDs (33). bsAb lutikizumab targeting IL-1 alpha and IL-1 beta was developed using the DVD-Ig technology platform for the treatment of synovitis in knee osteoarthritis and has been completed in OA (hand and knee OA) Two phase II trials were conducted.

#### 2.1.2 Non-IgG subtypes

This type of bsAb has no Fc region and is based on the structure of a single-chain variable fragment (scFv), which has the advantages of weak immunogenicity, low dosage, small size, and strong tissue penetration (34). The disadvantages are short half-life, low expression, and unstable structure (35). scFv is composed of antibody VL and VH connected by a short peptide (linker) of 15-20 amino acids, without Fc fragment, and belongs to small molecule genetically engineered antibodies. Linker is usually 15-20 amino acids in length, usually composed of glycine (Gly) and serine (Ser), and has certain elasticity and protease resistance. Technologies for preparing bsAbs with this structure include BiTE, DART, bi-Nanobody, TandAbs, etc., and only BiTE has been clinically validated.

##### 2.1.2.1 BiTE

**Figure 3:**
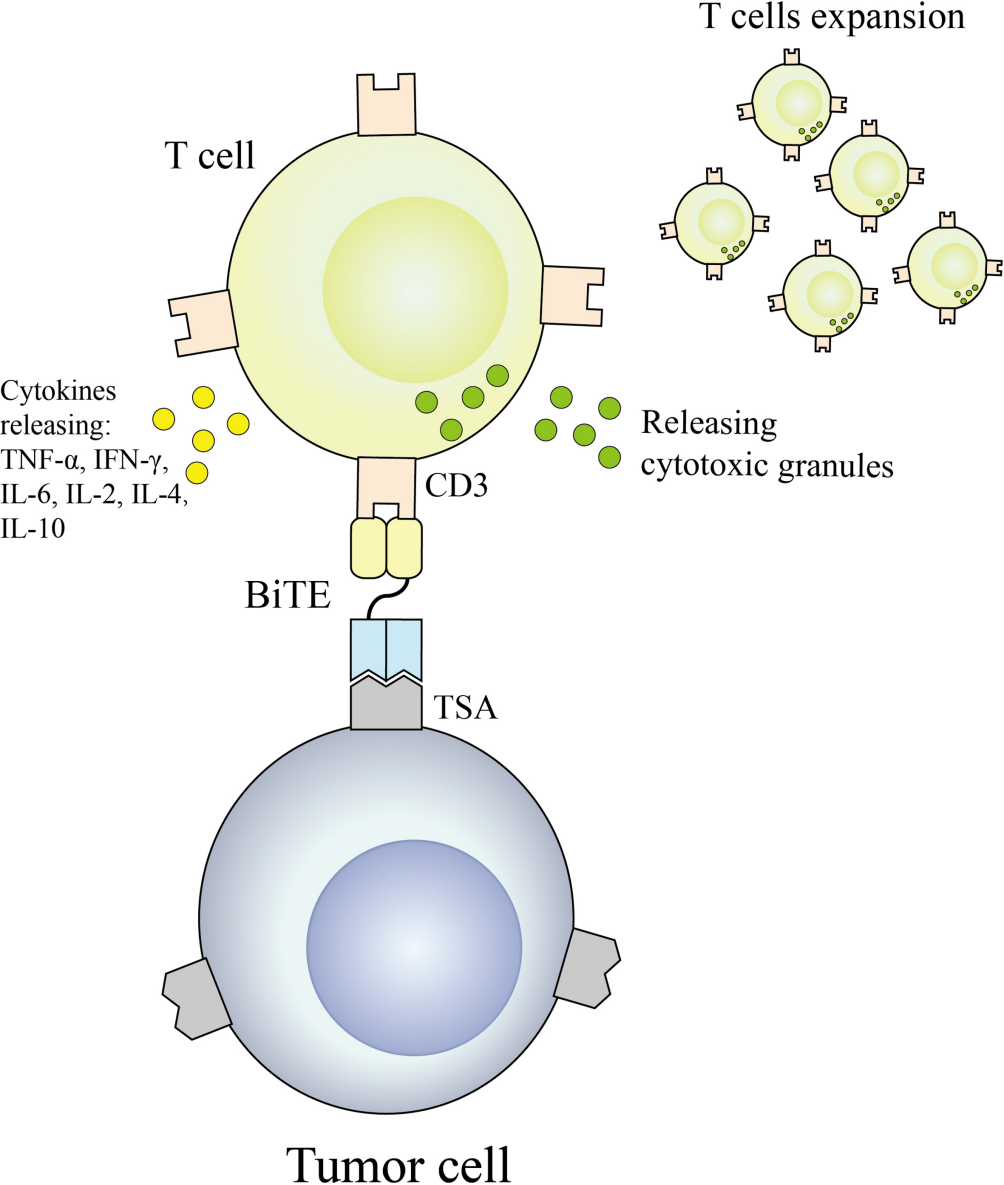
BiTE antibodies redirect T cells to a tumor cell.

BiTE is an antibody construct with 2 binding domains that recognize tumor-expressed antigens and CD3 of T cells, respectively, thereby linking endogenous T cells to tumor-expressed antigens, activating the cytotoxic potential of the patient’s own T cells to eliminate cancer without genetic modification or ex vivo expansion of T cells (36, 37). The binding domains are 2 scFv regions from a monoclonal antibody linked by a flexible peptide linker. The first scFv binding domain can be modified to target any surface antigen and thus can be targeted for different types of tumors. The second scFv binding domain was always specific for CD3 in the TCR. When BiTE molecules bind to both cytotoxic T cells and tumor cells, T cells begin to proliferate, thereby increasing the total number of effector cells and enhancing the effect of BiTE treatment (38), which then leads to tumor cell lysis. Because this does not require co-stimulation or the typical complex mechanisms of major histocompatibility, BiTE molecules can engage any T cell (39, 40). Blinatumomab is the first approved classical BiTE molecule, linking the variable regions of two monoclonal antibodies targeting CD19 on tumor cells and CD3 on T cells through a polypeptide chain, making blinatumomab largely Independent of genetic alterations or intracellular escape mechanisms (41). Because it is mainly composed of two single-chain antibodies, BiTE has a small molecular weight (55-60kDa), easily penetrates tumor tissue, and lacks the Fc segment, so its immunogenicity is low. Clinical trials have demonstrated that even at very low doses, blinatumomab can effectively recruit T cells and clear tumors, significantly improving median OS in patients with relapsed or refractory B-cell precursor acute lymphoblastic leukemia. The mechanism of BiTE is shown in **Figure 3**.

##### 2.1.2.2 DART

DART is developed by MacroGenics. DART molecules are heterodimers formed by the combination of two polypeptide chains, with two unique antigen-binding sites. Its structure is to use a linker to connect the VH and VL sequences of an antibody variable region with the VL and VH sequences of another antibody variable region to form scFv, and then co-express two scFv fragments, using the antibody VH and VL domains. interact to form bispecific fragments. In addition, cysteine was introduced at the C-terminus of the two polypeptide chains, and the interchain disulfide bond was formed through the cysteine, which improved the stability of the antibody (42). Unlike BiTE, DART is designed to mimic the natural interactions within IgG molecules, maintains potency both *in vitro* and *in vivo* administration, and aggregates at a lower scale in production. DART has better structural and biological properties than BiTE, including better stability and optimal redirection of the cytotoxic effect of T cells against malignant cells. The bsAb flotetuzumab targeting CD3ϵ and CD123 was developed based on the DART platform as a rescue immunotherapy for patients with refractory acute myeloid leukemia and is currently available in Japan and in Europe through an expanded access program (43).

##### 2.1.2.3 bi-Nanobody

The technology platform, developed by Ablynx (acquired by Sanofi in 2018), refers to the antibody structure of camelids (which only contains heavy chains and completely lacks light chains), and preserves the VH region through recombinant technology (44). Nanobody (Nanobody) is to connect the VH regions of two or more antibody molecules to achieve multi-specific binding. It has the advantages of small molecular weight, simple structure, strong tissue penetration, and can penetrate the blood-brain barrier. Sticking and clumping. But nanobodies also have the disadvantage of having a short half-life. In response to this problem, Ablynx uses half-life extension technology to combine nanobody with serum albumin, increase the molecular weight, and extend the half-life from several hours to more than 3 weeks with the help of the recycling effect of FcRn. Serum albumin can also play a role in transporting drugs to target sites. Ozoralizumab is a bi-Nanobody-based bsAb consisting of two anti-TNFα nanobodies and an anti-HSA nanobody. It is a good TNF inhibitor that inhibits arthritis progression (45) and is currently in phase II clinical studies for the treatment of rheumatoid arthritis.

##### 2.1.2.4 TandAbs

This technology platform, developed by Affimed, produces tetravalent bsAbs containing two peptide chains that provide two binding sites for each antigen (46). The N-terminus to C-terminus of each peptide chain is arranged in the order of VL1-VH2-VL2-VH1, and the two peptide chains are paired in opposite directions to form a homodimeric molecule. Such antibodies can recruit NK cells or T cells to the surrounding tumor, thereby harnessing the innate or adaptive immune system to fight cancer. The relative molecular mass of TandAbs is about 110 kD, which is between the whole molecule antibody and BiTE (about 50 kD), and the half-life of the product can reach 23 h. The protein has no glycosylation modification, the product is more uniform, and the immunogenicity is low. The bsAb AFM13, which targets CD30 and CD16A on NK cells, was developed based on this platform. CD30 is highly expressed in Hodgkin lymphoma, and CD16A is an active receptor involved in tumor cell killing (47). A Phase I clinical trial of AFM13 for the treatment of relapsed/refractory CD30-positive Hodgkin lymphoma has been completed, and results were presented at the American Association for Cancer Research 2021 Annual Meeting, demonstrating that AFM13 treats relapsed/refractory CD30-positive Hodgkin lymphoma The objective response rate of the tumor was 100%.

### 2.2 Mechanism of action

**Figure 4:**
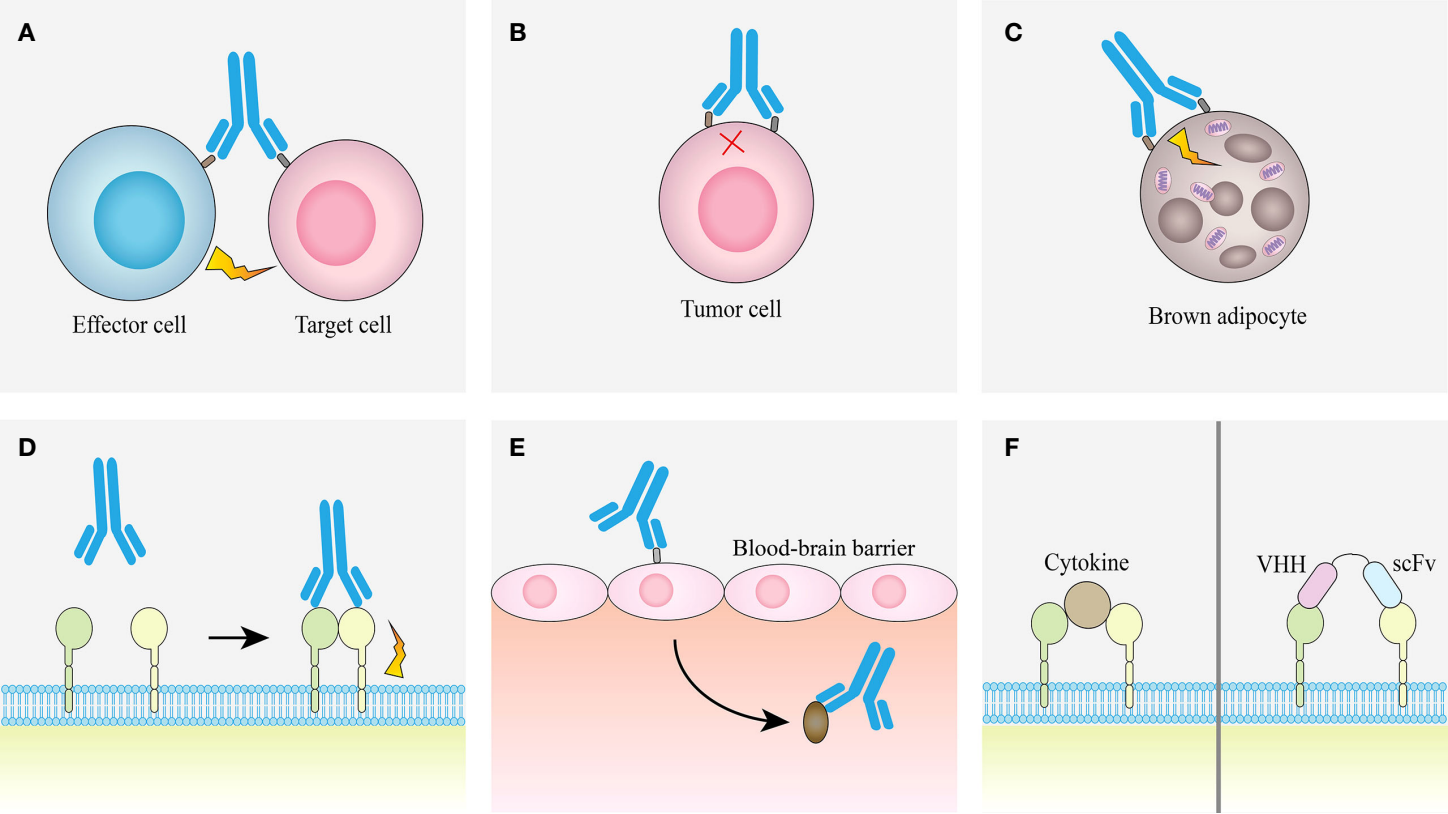
Examples of obligate mechanisms of action of bsAbs. **(A)** Linking T cells and tumor cells. **(B)** Receptor inhibition. **(C)** Receptor activation. **(D)** Analoging cofactor. **(E)** Using target to transport. **(F)** Surrogate cytokine agonists.

The advantage of bsAb is that it can bind two different epitopes at the same time, which provides a variety of different pathways for the realization of its function, so that it can treat diseases through different mechanisms (**Figure 4**).

#### 2.2.1 Linking T cells and tumor cells

This method mainly connects effector T cells and tumor cells through bsAb (48), also called T cell engaging bsAb (T-bsAb). The bsAb in this approach contains both a T-cell binding domain and a tumor-binding domain, which can bind to CD3ϵ in the CD3ϵ-TCR complex and activate T cells without antigen presentation (49) to kill tumor cells. However, only two T-bsAbs, catumaxomab and blinatumomab, are currently approved for clinical use, mainly due to difficulties in protein engineering during antibody preparation and the potential toxicity of the new constructs (50), as well as the clinically encountered association with cytokine release syndrome and neurotoxicity-related disorders (51, 52). However, studies have shown that T-bsAb has properties such as CD3 affinity screening and bystander effect, it can actively redirect internal T cells and promote their recruitment from the periphery to tumor tissues (53), and a variety of proteases accumulate in large quantities, which are beneficial for T cells and tumor cells. The formation of cytolytic synapses between target cells provides the necessary components (54). At the same time, T cells proliferate and release a variety of cytokines (55). Many mechanistic findings have been reported in preclinical studies, we are able to modulate the effects of T-bsAbs at the molecular or cellular level, and many clinical trials evaluating the efficacy and safety of T-bsAbs are ongoing. Therefore, T-bsAb is a promising emerging antibody therapy. The current state-of-the-art T-bsAb is a carcinoembryonic antigen T-cell bispecific antibody (CEA TCB) named cibisatamab (56).

##### 2.2.1.1 Cibisatamab

Cibisatamab has a single binding site for the CD3ϵ chain on T cells and two CEA binding sites that modulate binding affinity to cancer cells with moderate to high cell surface expression of CEA (21). The introduction of a CH1-CL cross into the CD3-bound Fab formed the light chain of cibisatamab (18), while the correct association of the heavy chain was achieved by the knob-into-hole technique (23, 57). The CEA-binding agent (designated CH1A1A) used in the CEA TCB is derived from the PR1A3 antibody and possesses properties such as humanization, affinity maturation, and stabilization (58, 59). CEA, also known as carcinoembryonic antigen–related cell adhesion molecule 5 (CEACAM5) or CD66e, is a 180 to 200 kDa protein belonging to the CEACAM superfamily that is anchored to the cell surface *via* glycosylphosphatidylinositol (GPI), It is often highly expressed in various tumor entities (60). Preliminary results of cibisatamab in a phase Ia/Ib study in locally advanced and/or metastatic CEA-positive solid tumors reported that monotherapy produced significant antitumor activity and manageable adverse events (61). The data demonstrate that CEA TCB has favorable antitumor activity and the ability to alter the tumor microenvironment, is effective against non-inflammatory, poorly invasive tumors, and transforms non-inflammatory tumors into highly inflammatory tumors.

#### 2.2.2 Receptor inhibition

Receptor tyrosine kinases (RTKs) are the largest class of enzyme-linked receptors, which are both growth factor receptors and enzymes that can catalyze the phosphorylation of downstream target proteins, including HER family, VEGFR family, etc. RTKs play an important regulatory role during cell proliferation, and precise regulation of the magnitude and duration of their signaling is critical for the execution of cellular programs and behaviors (62). Mutations in RTKs and their abnormal activation of intracellular signaling pathways may contribute to the development of cancer (63). Tumors can be treated by targeted inhibition of tumor receptor tyrosine kinases (RTKs), but other RTKs can escape from specific receptor inhibition and activate parallel signaling pathways, resulting in drug resistance. Therefore, bsAbs targeting multiple RTKs or their ligands can be developed to simultaneously block two or more signaling pathways to reduce tumor cell escape and overcome drug resistance. For example, zenocutuzumab is an ADCC-enhanced IgG antibody developed using Merus’ Biclonics technology and MeMo technology to target HER-2 and HER-3.

##### 2.2.2.1 Zenocutuzumab

Zenocutuzumab is a HER2×HER3 bispecific antibody that is effective in treating tumors driven by NRG1 gene rearrangements. NRG1 rearrangements are recurrent oncogenic drivers in solid tumors. NRG1 binds to HER3, leading to heterodimerization with other HER/ERBB kinases, increasing downstream signaling and tumorigenesis, and NRG1 induces phosphorylation of HER2 and HER3 when only HER2 and HER3 are expressed (64). The HER2-HER3 dimer may be the most carcinogenic heterodimer in the ERBB family (65). Zenocutuzumab inhibits HER3 and AKT phosphorylation, induces the expression of apoptosis markers, and inhibits tumor cell growth. Zenocutuzumab binds to NRG1 or NRG1 fusion protein and prevents HER3 from interacting with NRG1 or NRG1 fusion protein. This inhibition produced robust efficacy in preclinical models and durable responses in patients with few treatment options. Zenocutuzumab is a promising treatment option in development for patients with NRG1 fusion-positive cancers. Based on this proof-of-concept, a global multicenter Phase 1/2 clinical trial in NRG1 fusion-positive cancers has been initiated (66). The FDA has granted Fast Track designation to zenocutuzumab for the treatment of patients with metastatic solid tumors harboring an NRG1 gene fusion who have progressed after receiving standard therapy.

#### 2.2.3 Receptor activation

The treatment of some diseases requires activation of receptor signaling using agonistic antibodies, so bsAbs can also be used to activate multicomponent receptor complexes with receptors and coreceptors. For example, fibroblast growth factor 21 (FGF21) controls energy expenditure and nutrient metabolism by stimulating FGF receptor (FGFR) isoforms (1c, 2c, and 3c) that bind to the coreceptor Klothoβ (KLB) (67), the long-term use of recombinant FGF21 can improve the metabolic health of humans, but there is also a problem that the rapid molecular turnover limits the therapeutic effect. The bispecific anti-FGFR1/KLB agonist antibody BFKB8488A designed against it can treat obesity-related metabolic defects by specifically activating the FGFR1/KLB complex. Only when KLB is present on the cell surface, BFKB8488A enhances dimerization of the c-isoform of FGFR1 (FGFR1c) and stabilizes the interaction between FGFR1c and the extracellular domain of KLB protein. A clinical study in overweight human participants demonstrated that a single dose of BFKB8488A resulted in transient weight loss, sustained improvement in cardiometabolic parameters, and a trend toward reduced preference for sweetness and carbohydrate intake (68). A phase II clinical study of BFKB8488A in nonalcoholic steatohepatitis is ongoing.

#### 2.2.4 Analoging cofactor

BsAbs can also mimic enzymes or cofactors in enzyme-substrate complexes. For example, hemophilia A is a bleeding disorder caused by a deficiency of factor VIII (FVIII), which normally acts as a cofactor for activating factor IX (FIXa) and promotes the activation of factor X (FX). The researchers designed bsAb(Mim8) as an activated FVIII mimetic. Mim8 is an anti-FIXa/anti-FX bispecific antibody whose activity is achieved in part through FIXa-stimulating activity present in the anti-FIXa arm. Preclinical experiments have shown that it has an effective hemostatic effect (69), and it has obtained an implied license for clinical trials in China. Intended to be developed for routine prophylaxis in children (<12 years of age) with hemophilia A (congenital factor VIII deficiency) with or without FVIII inhibitors to prevent or reduce the frequency of bleeding.

#### 2.2.5 Using target to transport

A bsAb can also utilize the specificity of its first target to transport its second specific target. For example, the blood-brain barrier is a huge obstacle to developing treatments for neurological diseases. The application of bsAbs to the transferrin transport pathway can cross the blood-brain barrier and enter the immune-privileged brain regions to target pathogenic mediators that cause neurological diseases (70). By targeting the transferrin receptor (TfR), which is highly expressed on the surface of brain endothelial cells, with one of the binding arms of a bsAb, the bsAb binds to TfR and crosses the blood-brain barrier into the brain through receptor-mediated endocytosis. The second binding arm targets beta-secretase 1 (BACE1), blocking BACE1 activity in the brain. TfR×BACE1 bsAb reduces the levels of amyloid-β (Aβ) peptide, an enzymatic product of BACE1, in brain tissue and cerebrospinal fluid (71). The amyloid-β (Aβ) peptide is thought to be involved in the pathogenesis of Alzheimer’s disease, and its pathological overproduction leads to impaired memory, oxidative damage, blood-brain barrier damage, neurofibrillary tangles, and amyloid plaque formation (72).

#### 2.2.6 Surrogate cytokine agonists

Cytokines can initiate signal transduction through receptor dimerization for immunomodulation, but their structure often influences their effects. BsAbs can dimerize cytokine receptors, thereby acting as a substitute for cytokines. Combining VHH and scFv can generate single-chain dual-specific ligands with multiple functional activities that force cytokine receptor heterodimerization and then initiate signaling. Type I IFNs have antiviral activity and can dimerize IFNAR1/IFNAR2 to activate certain signal transduction. Combining a single variable domain of a heavy chain (VHH) and a single-chain antibody fragment (scFv) in a type I IFN system to construct a surrogate agonist applied to human IFNAR1 and IFNAR2, which effectively inhibits viral replication, but does not have pro-inflammatory effects. This means that the strategy can obtain specific ligands corresponding to each receptor through screening, and it also means that the construction of dual-specific ligands is not limited to the induction of dimerization of natural receptors (73).

## 3 Application

The research and development of antibody drugs has always been a hot topic, among which bsAb is also a key target of corporate research and development. So far, a total of 7 bsAbs have been approved for marketing in the world, they are catumaxomab (which has been withdrawn from the market in 2017), blinatumomab, emicizumab, amivantamab, faricimab, candonilimab and mosunetuzumab (**Table 2**), and more than 200 bsAbs are in clinical and preclinical research stages (**Tables 3**, **4**).

**Table 2:** BsAbs that have been on the market.

	Company	Trade name	Common name	Time of approval	Target	Indication
1	TrionPharma	Removab	Catumaxomab	2009.4	CD20×EpCAM	Malignant ascites
2	Amgen	Blincyto	Blinatumomab	2014.12	CD3×CD19	ALL
3	Roche	Hemlibra	Emicizumab	2017.11	FIX×FX	Hemophilia A
4	Janssen	Rybrevant	Amivantamab	2021.5	EGFR×c-MET	NCSLC
5 6 7	Genentech Roche Akeso	Vabysmo Lunsumio -	Faricimab Mosunetuzumab Candonilimab	2022.1 2022.6 2022.6	VEGF×Ang-2 CD20×CD3 PD-1×CTLA-4	DME, w-AMD R/R FL R/M CC

**Table 3:** Bispecific antibodies in clinical trials.

Name	Target	Disease	Trial	Sponsor
A-319	CD19×CD3	B-cell malignancy	Phase I	Generon
ABL501	PD-L1×LAG-3	Advanced solid tumor	Phase I	ABL Bio
AFM11	CD19×CD3	Non-Hodgkin’s lymphoma	Phase I	Affimed
AK112	PD-1×VEGF	Extensive stage small cell lung cancer	Phase I	Akeso
AMG330	CD33×CD3	Acute myelogenous leukemia	Phase I	Amgen
AMG420	BCMA×CD3	Multiple myeloma	Phase I	Amgen
AMG424	CD38×CD3	Multiple myeloma	Phase I	Amgen
AMG562	CD19×CD3	Lymphoma	Phase I	Amgen
AMG673	CD33×CD3	Acute myelogenous leukemia	Phase I	Amgen
AMG701	BCMA×CD3	Multiple myeloma	Phase I	Amgen
AMV-564	CD33×CD3	Acute myelogenous leukemia	Phase I	Amphivena Therapeutics
APVO436	CD123×CD3	Acute myelogenous leukemia	Phase I	Amphivena Therapeutics
AZD2936	TIGIT×PD-1	Non-small-cell lung carcinoma	Phase I/II	AstraZeneca
AZD7789	PD-1×TIM-3	Non-small-cell lung carcinoma	Phase I/II	AstraZeneca
CDX-527	PD-L1×CD27	Solid tumors	Phase I	Celldex Therapeutics
Elranatamab	BCMA×CD3	Relapsed or refractory multiple myeloma	Phase I	Pfizer
EM801	BCMA×CD3	Multiple myeloma	Phase I	Celgene
EMB-02	PD-1×LAG-3	Advanced solid tumors	Phase I/II	Shanghai EpimAb Biotherapeutics Company
ES101	PD-L1×4-1BB	Solid tumors	Phase I	Elpiscience Biopharma
FBTAO5	CD20×CD3	B-cell lymphoma	Phase I/II	Trion
FS118	LAG-3×PD-L1	Advanced cancer	Phase I/II	F-star Therapeutics Limited
GBR 1342	CD38×CD3	Multiple myeloma	Phase I/II	Glenmark Pharmaceuticals
GEM333	CD33×CD3	Acute myelogenous leukemia	Phase I	GEMoaB Monoclonals
GEN3O13	CD20×CD3	B-cell non-Hodgkin lymphoma	Phase I/II	Genmab
HLX301	TIGIT×PD-L1	Locally advanced or metastatic solid tumors	Phase I/II	Shanghai Henlius Biotech
HX009	CD47×PD-1	Relapsed/refractory lymphoma	Phase I/II	Waterstone Hanxbio Pty Ltd
JNJ-64007957	BCMA×CD3	Multiple myeloma	Phase I	Janssen
KM257	HER2×HER2	Advanced solid tumors	Phase I	Xuanzhu Biopharmaceutical Company
MCLA-117	CLEC12A×CD3	Acute myelogenous leukemia	Phase I	Merus
MCLA-128	HER2×HER3	Solid tumors	Phase I/II	Merus
MCLA-129	EGFR×c-MET	Solid tumors	Phase I/II	Merus
MCLA-145	PD-L1×CD137	Solid tumors	Phase I	Merus
MDX447	EGFR×FcγRI	Brain and central nervous system tumors	Phase I	Dartmouth-Hitchcock Medical Center
MGD001	CD19×CD3	B-cell lymphoma	Phase II	MacroGenics
MGD006	CD123×CD3	Acute myeloid leukemia	Phase I/II	MacroGenics
Nivatrotamab	GD2×CD3	Small-cell lung cancer	Phase I/II	Y-mAbs Therapeutics
Odronextamab	CD20×CD3	B-cell non-Hodgkin lymphoma	Phase II	Regeneron Pharmaceuticals
PF-06863135	BCMA×CD3	Multiple myeloma	Phase I	Pfizer
Plamotamab	CD20×CD3	Hematologic malignancy	Phase I	Xencor
PRS-344/S095012	PD-L1×4-1BB	Solid Tumor	Phase I/II	Pieris Pharmaceuticals
REGN1979	CD20×CD3	B-cell non-Hodgkin’s lymphoma	Phase II	Regeneron Pharmaceuticals
REGN4336	PSMA×CD3	Metastatic castration-resistant prostate cancer	Phase I/II	Regeneron Pharmaceuticals
REGN5458	BCMA×CD3	Multiple myeloma	Phase I	Regeneron Pharmaceuticals
RG6026	CD20×CD3	Non-Hodgkin’s lymphoma	Phase I	Roche
RO7121661	PD-1×TIM-3	Solid tumors	Phase I	Hoffmann-La Roche
RO7247669	PD-1×LAG3	Solid tumors	Phase I	Hoffmann-La Roche
SI-B001	EGFR×HER3	Locally advanced or metastatic epithelial tumor	Phase I	Sichuan Baili Pharmaceutical Company
SI-B003	PD-1×CTLA-4	Solid tumors	Phase I	Sichuan Baili Pharmaceutical Company
Talquetamab	GPRC5D×CD3	Hematological malignancies	Phase II	Janssen Research & Development
Teclistamab	BCMA×CD3	Hematological malignancies	Phase I	Janssen Research & Development
TG-1801	CD19×CD47	B-cell lymphoma	Phase I	TG Therapeutics
TNB-383B	BCMA×CD3	Multiple myeloma	Phase I	AbbVie
TNB-486	CD19×CD3	Lymphoma	Phase I	TeneoTwo
TNB-585	PSMA×CD3	Metastatic castration-resistant prostate cancer	Phase I	Amgen
Xmabl4045	CD123×CD3	Chronic myelogenous leukemia	Phase I	Xencor
Y101D	PD-L1×TGF-β	Metastatic or locally advanced Solid Tumors	Phase I	Wuhan YZY Biopharma Company
ZW25	HER2×HER2	Solid tumors	Phase I/II	BeiGene

**Table 4:** BsAbs in clinical trials in combination with other immunotherapy.

Name	Target	Immunotherapy	Disease	Trial	Sponsor
AK112	PD-1×VEGF	AK117	Advanced malignant tumors	Phase I/II	Akeso
		Etoposide, Carboplatin	Extensive stage small cell lung cancer	Phase I	
Elranatamab	BCMA×CD3	Nirogacestat	Multiple myeloma	Phase II	Pfizer
INBRX-105	PDL1×41BB	Pembrolizumab	Solid tumors	Phase I	Inhibrx
KN046	PD-L1×CTLA-4	Lenvatinib	Advanced hepatocellular carcinoma	Phase II	Beijing Cancer Hospital
REGN5458	BCMA×CD3	Cemiplimab	Multiple myeloma	Phase I/II	Regeneron Pharmaceuticals
RO6958688	CEA×CD3	Obinutuzumab, Tocilizumab,	Solid tumors	Phase I	Hoffmann-La Roche
TF2	CEA×hapten	Lu-177-labeled IMP-288	Colorectal neoplasms	Phase I	Radboud University Medical Center
TG-1801	CD19×CD47	Ublituximab	B-cell lymphoma	Phase I	TG Therapeutics
Vibecotamab	CD3×CD123	Dexamethasone, Acetaminophen, Diphenhydramine	Acute myeloid leukemia	Phase II	M.D. Anderson Cancer Center
XmAb18087	SSTR2×CD3	Pembrolizumab	Merkel Cell Carcinoma	Phase I/II	Xencor
XmAb^®^22841	LAG-3×CTLA-4	Pembrolizumab		Phase I	Xencor
ZW25	HER2×HER2	Docetaxel	Breast cancer	Phase I/II	BeiGene
		Tislelizumab, Capecitabine, Oxaliplatin			

### 3.1 BsAbs that have been on the market

#### 3.1.1 Catumaxomab

Catumaxomab is the world’s first commercialized monoclonal bispecific trifunctional antibody, developed by TrionPharma, produced by Triomab quadroma technology, and approved by EMA in April 2009 for intraperitoneal use in patients with malignant ascites caused by EpCAM-positive tumors treatment, but was withdrawn from the market in 2017 due to, among other reasons, poor sales. Catumaxomab consists of mouse IgG2 and rat IgG2b and targets CD3 on T cells and EpCAM on tumor cells (74). Epithelial cell adhesion molecule (EpCAM) is a 40KD type I transmembrane glycoprotein consisting of an extracellular domain (EpEX), a single transmembrane domain and an intracellular domain (EpICD) (75). Studies have shown that it plays an important role in intercellular adhesion, cell signaling, proliferation, differentiation, and the formation and maintenance of organ morphology (76). EpCAM is expressed on the entire cell surface (77) and is a very strongly and frequently expressed tumor-associated antigen (78), in ovarian, gastric, colon, pancreatic, prostate, lung and endometrial cancers. There are expressions (79). Although catumaxomab has been withdrawn from the market in 2017, its related research has not stopped. Recent studies have shown that two patients with EpCAM-positive recurrent non-muscle invasive bladder cancer were first treated with intravesical catumaxomab, which was well tolerated without any overt signs of toxicity and was Intravesical administration of catumaxomab is feasible, safe, and effective in muscle-invasive bladder cancer, thus supporting its further clinical development in this indication (80). Catumaxomab is currently undergoing clinical trials in breast, ovarian, peritoneal, non-small cell lung and gastrointestinal cancers (81).

#### 3.1.2 Blinatumomab

Blinatumomab was developed by Amgen and was approved by the FDA in December 2014 for the treatment of Fisher chromosome-negative precursor B-cell acute lymphoblastic leukemia. Blinatumomab is a bispecific monoclonal antibody derived from recombinant mouse. It is a class of bispecific T cell engagers (BiTEs) that target the CD3 cell surface antigen on T lymphocytes and the CD19 locus on (malignant) B lymphocytes (54), enabling T cell trafficking to tumor cells And showed strong tumor killing ability. In Phase I/II clinical trials, blinatumomab was demonstrated in patients with relapsed and/or refractory (R/R) non-Hodgkin lymphoma and R/RB cell precursor acute lymphoblastic leukemia (B precursor ALL) Significant single drug activity. Cytokine release syndrome and neurologic side effects are both dose-limited, with manageable and transient adverse effects (82). However, blinatumomab also has the characteristics of possible drug resistance (83) and easy renal clearance due to its small size (84). Recent studies have shown that Among children with high-risk first-relapse B-cell acute lymphoblastic leukemia, treatment with 1 cycle of blinatumomab compared with standard intensive multidrug chemotherapy before allogeneic hematopoietic stem cell transplant resulted in an improved event-free survival at a median of 22.4 months of follow-up (85). The experimental results prove that blinatumomab has a good market prospect.

#### 3.1.3 Emicizumab

Hemophilia A (HA) is a congenital X-linked bleeding disorder that results in complete or partial loss of activated factor VIII (FVIII) coagulation activity. Emicizumab is a recombinant humanized bispecific monoclonal antibody that restores the function of missing FVIII by bridging FIXa and FX to promote effective hemostasis in hemophilia A (HA) patients (86). Emicizumab mimics the cofactor activity of activated FVIII (FVIIIa), one arm of which binds to epidermal growth factor (EGF)-like domain 1 of FIX/FIXa, and the other arm recognizes EGF-like domain 2 of FX/FXa (87), thereby bridging FIXa and FX as a cofactor to form a sterically correct structure that facilitates the conversion of FX to FXa. Emicizumab is only functionally similar to FVIII but has a completely different molecular structure and does not bind to existing FVIII antibodies, so it is effective even in the presence of FVIII antibodies and does not induce the production of new FVIII antibodies (88). Emicizumab was developed by Roche and was approved by the FDA in November 2017 for the treatment of HA. Studies have shown that emicizumab prophylaxis is associated with a significantly lower rate of bleeding events compared with no prophylaxis in HA participants using inhibitors (89); in HA patients not using inhibitors, once a week or every 2 Once-weekly subcutaneous emicizumab prophylaxis was associated with significantly lower bleeding rates compared with no prophylaxis; more than half of the participants who received prophylaxis had no treatment-bleeding events (90). This shows that the therapeutic effect of emicizumab in HA is quite effective and has a good market prospect.

#### 3.1.4 Amivantamab

Amivantamab, a bsAb targeting EGFR and MET (91), was developed by Janssen Biotechnology and approved by the FDA in May 2021 for patients with non-small cell lung cancer (NSCLC) whose tumors have *EGFR* exon 20 insertion mutations (92). Aberrant activation of *EGFR* and *MET* signaling pathways drives tumor cell growth and proliferation in lung cancer (93–96). EGFR mutations become constitutively activated signaling pathways that generate pro-survival and anti-apoptotic signals. Cancer cells with *EGFR* mutations become dependent on EGFR for survival, making EGFR an attractive target for anticancer therapy (97). *c-MET* amplification activates parallel oncogenic signaling pathways that bypass *EGFR*, resulting in resistance to EGFR-KI (98). Amivantamab binds to the extracellular domains of EGFR and cMet and blocks the binding of the ligand EGF to EGFR and the binding of the ligand HGF to its receptor cMet; it also induces degradation of both receptors *in vivo*, extending its effects to Ligand-independent receptor-driven diseases are included (99), and can bind to immune effector cells through ADCC to eliminate antigen-expressing tumor cells (100). The study showed that in patients with locally advanced or metastatic NSCLC with EGFR exon 20 insertion mutation after platinum-based chemotherapy, the overall response rate after using amivantamab was 40%, and the median duration of response was 11.1 months, indicating a good clinical effect (101).

#### 3.1.5 Faricimab

Faricimab, which targets VEGF-A and Ang-2, was developed by Genentech and approved by the FDA in January 2022 for the treatment of wet age-related macular degeneration (wAMD) and diabetic macular edema (DME), the first approved bsAbs in this field. VEGF is an important cytokine in angiogenesis (102), and VEGF-A in particular is the target of current retinal diseases. It binds to the extracellular ligand-binding domains of VEGFR-1 and VEGFR-2, thereby activating internal signaling pathways that alter angiogenesis and vascular permeability genes (103). The Tie-2/Angiopoietin pathway plays an important role in vascular development and function (104). Activation of Tie-2 receptors by Ang-1 can maintain vascular stability to limit extravasation. Ang-2 replaces Ang-1 by binding to Tie-2 receptors and prevents its activation, thereby blocking the anti-inflammatory and anti-inflammatory effects of Ang-1. Apoptosis and tight binding support (105). Co-expression of Ang-2 and VEGF-A accelerated neovascularization in the developing retina and in ischemic retinal models. Studies have shown that Ang-2 and VEGF levels are elevated in vitreous samples from diabetic patients (106). Serum VEGF and Ang-2 levels were also higher in the proliferative diabetic retinopathy group than in the non-proliferative diabetic retinopathy group, suggesting that their levels may be associated with the progression of retinopathy (107). Preliminary 1-year results from Phase 3 trials of YOSEMITE and RHINE suggest that faricimab, which inhibits the dual action of Ang-2 and VEGF-A pathways, is designed for intraocular use to provide non-deteriorating visual gain and improved anatomical outcomes that can be achieved by This is achieved with dose adjustments up to every 16 weeks (108).

#### 3.1.6 Mosunetuzumab

Mosunetuzumab was developed by Roche and approved by the FDA in June 2022. It is a CD20×CD3 T-cell engaging bsAb which can be used to treat adult patients with relapsed/refractory follicular lymphoma (R/R FL) who have received at least two prior systemic therapies (109). It is also the world’s first listed CD20×CD3 bsAb. CD3×CD20 is currently one of the most competitive bsAb target combinations. This type of bsAb can simultaneously bind to CD20 expressed on the surface of many B cell malignant tumors and CD3 on the surface of immune T cells, which can recruit T cells to surrounding tumor cells and activate T cells to kill tumor cells, providing tumor patients with an innovative treatment. In clinical trials, mosunetuzumab demonstrated high complete response rates, with most patients lasting at least 18 months of remission after complete responses, and was well tolerated in FL patients who had received extensive frontline therapy. A clinical development program for mosunetuzumab is underway to study the molecule as a monotherapy and in combination with other agents in patients with B-cell non-Hodgkin lymphoma, including follicular lymphoma, diffuse large B-cell lymphoma and other blood cancers.

#### 3.1.7 Candonilimab

Candonilimab is a bsAb which targets PD-1 and CTLA-4, developed by Akeso and approved by NMPA in June 2022, for the treatment of R/M CC after failure of platinum-based chemotherapy. It is the first dual immune checkpoint inhibitor bsAb approved for marketing in the world. The median overall survival of patients with recurrent or metastatic cervical cancer treated with candonilimab was 17.51 months, and the overall survival of patients was prolonged by 8-13 months. At present, the phase III clinical study of candonilimab plus platinum-based chemotherapy with or without bevacizumab in the first-line treatment of persistent, recurrent or metastatic cervical cancer has been completed. Phase III pivotal registrational clinical study of primary adenocarcinoma, registrational/phase III clinical study of candonilimab combined with concurrent chemoradiotherapy for locally advanced cervical cancer, and the efficacy of candonilimab for adjuvant therapy after radical resection of hepatocellular carcinoma with high recurrence risk. Phase III clinical studies are in progress.

### 3.2 Application of bsAb in tumor immunotherapy

Over the past two decades, tumor immunotherapy has developed into the fourth pillar of cancer treatment in addition to surgery, radiotherapy and chemotherapy (110). One of the most successful immunotherapy modalities is antibody therapy (111). Among the 119 clinical and 176 preclinical bsAb projects, there are 99 clinical and 153 preclinical projects for tumor therapy. The roles of bsAb in tumor treatment are as follows.

#### 3.2.1 Anti-angiogenesis

Angiogenesis, the formation of new blood vessels from pre-existing blood vessels, is one of the hallmarks of cancer (112). Tumor cells develop an angiogenic phenotype such that pro-angiogenic mechanisms overwhelm anti-angiogenic mechanisms. Endothelial cells grow rapidly, enabling oxygen and nutrients to reach the tumor microenvironment, supporting tumor growth and spread (113, 114). Identified pro-angiogenic molecules include VEGF, transforming growth factor-α and -β (TGF-α and -β), epidermal growth factor and so on (115). In addition, bsAb can block two or even multiple angiogenesis pathways at the same time, enhancing the anti-angiogenic effect. For example, bsAb ABL001 targeting vascular endothelial growth factor (VEGF) and Delta-like ligand4 (DLL4) competitively inhibits VEGF binding to VEGFR-2 and DLL4 to Notch1, blocking VEGF/VEGFR2 and DLL4/Notch1 in endothelial cells signaling pathway that inhibits angiogenesis. Compared with antibodies targeting VEGF alone and antibodies targeting DLL4 alone, ABL001 has shown higher potency anticancer effects in several human cancer xenograft models (116, 117), and is currently being evaluated in a phase 1 clinical study of heavy chemotherapy or targeted therapy pre-treated cancer patients (118).

#### 3.2.2 Anti-tumorigenesis

There are high or abnormal expression of cytokine receptors in many tumors, resulting in an increase in the number of receptors or constant activation, which may lead to enhanced downstream signaling and uncontrollable cell proliferation, thereby causing cancer. Targeting oncogenic receptors is one of the widely used antitumor approaches. Most of the targeted sites of these bsAbs are HER2, HER3, EGFR, cMet, and LRP5/6. Zanidatamab is a bsAb developed by Zymeworks that can simultaneously bind two non-overlapping epitopes of HER2, ECD2 and ECD4 (119), called biparatopic binding. This unique design results in multiple mechanisms of action, including dual HER2 signaling blockade, increased cell surface HER2 protein binding and removal, and potent effector functions that lead to enhanced antitumor activity. Zymeworks is currently conducting multiple Phase 1, 2 and pivotal clinical trials worldwide to develop zanidatamab as a targeted therapy option for patients with HER2-expressing solid tumors. 1G5D2 is a bsAb targeting HER2 and HER3, and it can specifically bind to HER2 and HER3 expressed on tumor cells, thereby inhibiting AKT and ERK downstream signaling pathways and tumor cell proliferation *in vitro* (120), indicating that it may act as a HER2/HER3 transducer. A therapeutic candidate for expressing cancer is still in preclinical research.

#### 3.2.3 Enhance T cell function

Tumor immunotherapy aims to improve the patient’s own anti-tumor immune response. It can enhance anti-tumor immunity by enhancing T cell helper activation signals. Common target sites include 4-1BB and OX40. Researchers developed a bsAb targeting B7-H3 and 4-1BB, which enhanced the proliferation and cytokine production of terminally differentiated CD8+ tumor-infiltrating lymphocytes, thereby enhancing antitumor immunity (121). B7-H3 (CD276) is a tumor antigen that is overexpressed in various human malignancies and tumor-associated vasculature, but not in healthy tissues. 4-1BB (CD137) is a costimulatory receptor that is widely expressed on immune cells and belongs to the tumor necrosis factor (TNF) receptor superfamily. It-mediated T cell co-stimulation can enhance T cell proliferation, cytotoxicity, and cytokine secretion, and protect T cells (122). Compared with the 4-1BB agonist mAb, the B7-H3 × 4-1BB bsAb showed more potent T-cell co-stimulatory activity and higher tumor localization *in vitro*. The bsAb is currently in the preclinical research stage.

#### 3.2.4 Remove T cell inhibition

Boosting anti-tumor immunity can also be achieved by blocking T cell response inhibitory signals, commonly targeting sites including CTLA-4 and PD-1/PD-L1. We developed ATOR-1015, an IgG1 bsAb targeting OX40 and CTLA-4, that induces T cell activation and regulatory T cell (Treg) depletion *in vitro*. CTLA-4 is constitutively expressed on Tregs and is highly expressed in the tumor microenvironment, especially in Tregs (123). OX40 (CD134) is a co-activating member of the NGFR/TNFR superfamily expressed on activated CD4+ and CD8+ T cells, neutrophils and natural killer cells. OX40 has an important costimulatory function during T cell activation, mediating the survival and expansion of CD4+ and CD8+ T cells in a variety of animal models of autoimmunity, infectious diseases, and cancer (124). OX40 is also involved in the control of effector and memory T cell responses (125). ATOR-1015 reduces tumor growth and improves survival in several syngeneic tumor models, including bladder, colon, and pancreatic cancer models; also induces tumor-specific and long-term immune memory and enhances response to PD-1 inhibition. ATOR-1015 localizes to the tumor area, reduces the frequency of Tregs and increases the number and activation of CD8+ T cells (126). Currently, ATOR-1015 has completed the first-in-human Phase 1 clinical trial in patients with advanced and/or refractory solid malignancies.

#### 3.2.5 Modulating tumor microenviroment

To escape immune system surveillance, tumors can suppress the function and proliferation of immune cells in the tumor microenvironment (TME) by expressing immunosuppressive molecules (eg, TGFβ, CD73) and recruiting immunosuppressive cells. Some bsAbs were developed to overcome immunosuppressive effects in the TME. For example, programmed cell death protein 1 (PD-1), an immune checkpoint expressed on T cells, inhibits T cell activity when it binds to the ligands PD-L1 or PD-L2. The PD-1-PD-L1 axis is not only an important feedback loop for immune homeostasis, but also involved in tumor immune escape (127). Transforming growth factor-β (TGF-β) is a tumor suppressor that inhibits cell proliferation and inflammation, and induces apoptosis. TGF-β is overexpressed in advanced tumors and is associated with poor prognosis (128). In TMEs with hyperactive TGF-β signaling, anti-PD-1/PD-L1 therapy has limited efficacy (129), necessitating simultaneous blockade of both PD-1/PD-L1 and TGF-β pathways. The researchers developed a bsAb YM101 targeting TGF-β and PD-L1, the world’s first anti-PD-L1 and TGF-β bispecific antibody. It can block both TGF-β and PD-L1 pathways simultaneously, and has a good antitumor effect (130), and *in vivo* experiments showed that the antitumor activity of YM101 was superior to that of anti-TGF-β and anti-PD-L1 monotherapy. YM101 promotes immune-inflammatory tumor formation, normalizes dysregulated antitumor immunity, and provides an immune-supportive TME. Y101D was granted an implied clinical license on May 17, 2021 for locally advanced or metastatic tumors.

#### 3.2.6 Depletion of target cell

This type reflects the biological mechanism of most of the bsAbs currently under development, that is, by mediating effector cells or through Fc-mediated functions such as ADCC, antibody-dependent celluar phagocytosis (ADCP) and CDC, it directly targets tumor cells to achieve the purpose of clearance. Catumaxomab works through this mechanism. It uses the Triomab molecular pattern and relies on two antigen-binding arms to bind the CD3 site of cytotoxic T cells and the EpCAM site of tumor cells respectively, thereby guiding T cells to kill target cells (131–133). The bsAb AFM24 developed by Affimed targets EGFR and CD16A located on innate immune cells and can bind and redirect innate immune cells such as NK cells and macrophages to EGFR^+^ tumor cells (134), thereby directly killing tumor cells. AFM24 is effective against a variety of EGFR-expressing tumor cells regardless of EGFR expression level and KRAS/BRAF mutation status (135). AFM24 is currently in a Phase II clinical study for advanced solid tumors.

## 4 Challenges in the future

**Figure 5:**
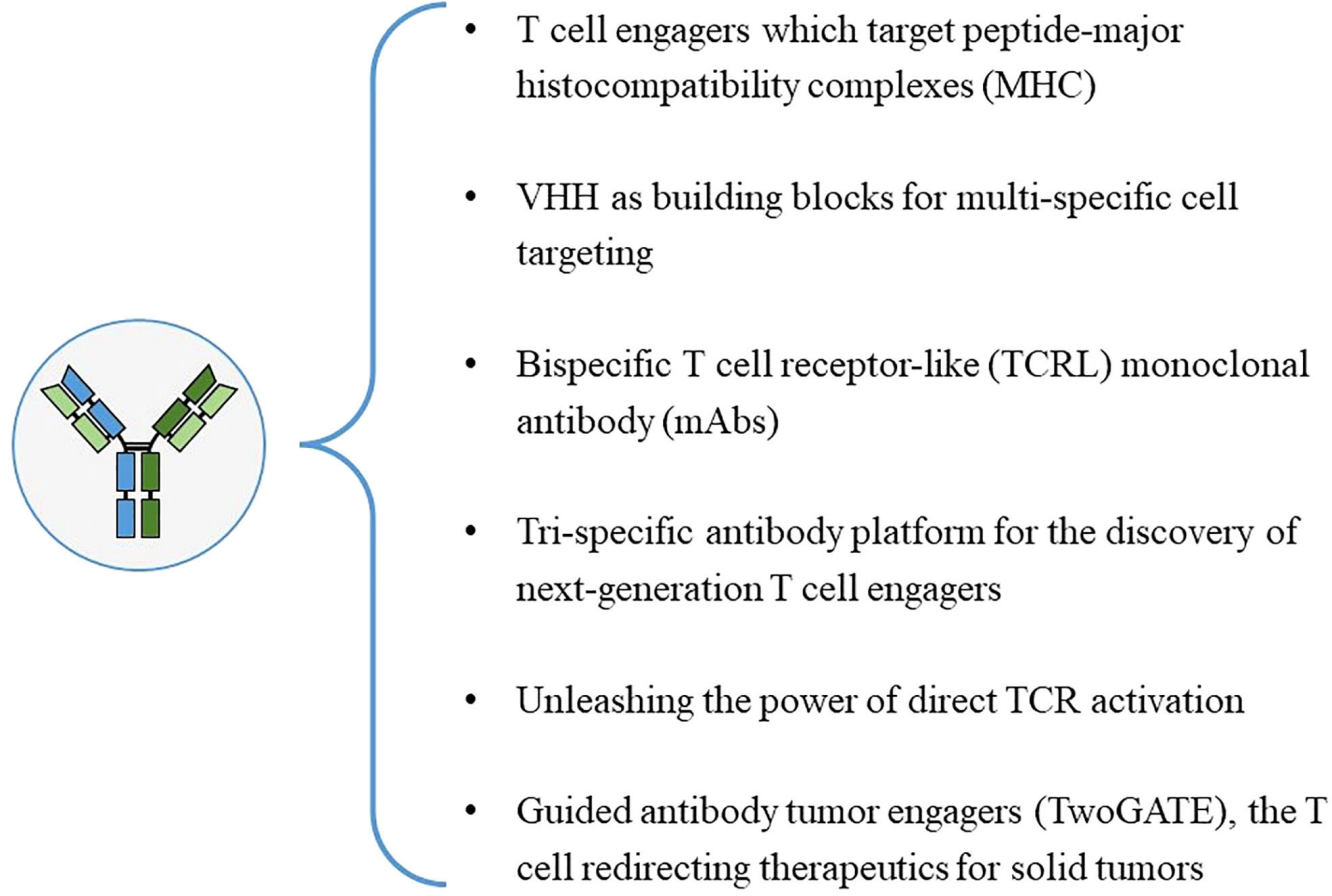
The future directions of bsAbs.

At this stage, the research and development of antibody drugs has a good momentum of development, and the research in the field of bsAb is also in full swing (**Figure 5**). However, there are also some risks in the clinical application of bsAbs. The higher affinity of bsAbs for CD3 may lead to activation of T cells by bsAbs before they bind to tumor cells, inducing a large release of cytokines and triggering a cytokine storm. In addition, the Fc fragment of bsAbs is immunogenic and can bind to Fc receptors on the surface of other effector cells, so full-length double antibodies are more likely to cause tumor-independent T cell activation, and in extreme cases, cytokine storms. Furthermore, the molecular structure of bsAbs does not exist in nature, which enhances the immunogenicity and elicits an immune response in the body. Therefore, the structure of bsAbs should be designed with a reasonable range of antibody affinity, which can inhibit Fc-mediated effector function as much as possible while having a more specific tumor target. Improving the humanized components of bsAbs and evaluating the immunogenicity in preclinical and clinical trials will effectively improve the safety and clinical efficacy of bsAbs.

## 5 Conclusions

The rapid development of antibody drugs has promoted the development of bsAbs. So far, more than 200 bsAbs are in clinical and preclinical research stages, and there are 7 bsAbs have been approved for marketing in the world. BsAbs have diverse structures, have different mechanisms of action, and are widely used in the treatment of tumors and other diseases. In the field of tumor therapy, bsAbs are widely used, and play a number of roles in anti-angiogenesis, regulation of signaling pathways, and regulation of tumor microenvironment. At present, most bsAbs are still in the clinical or preclinical research stage, and there is still a certain distance from the market. In the process of research and development, they still face many problems. With the advancement of science and technology, the research on bsAb will be further developed. It is believed that many problems faced in the research and development process will be gradually solved. We also expect that more and more bsAbs will be used in clinical treatment in the future to treat more patients with pain.

## Author contributions

JK and TS: conceptualization, methodology, software, investigation, formal analysis, and writing - original draft; YZ: conceptualization, funding acquisition, resources, supervision, and writing - review and editing. All authors contributed to the article and approved the submitted version.

## Funding

The study was funded by the 1·3·5 project for disciplines of excel-lence-Clinical Research Incubation Project, West China Hospital, Sichuan University (Grant No. 2019HXFH062) and from the Key R&D Projects of the Science and Technology Department of Sichuan Province (No. 2021YFS0237)

## Conflict of interest

The authors declare that the research was conducted in the absence of any commercial or financial relationships that could be construed as a potential conflict of interest.

## Publisher’s note

All claims expressed in this article are solely those of the authors and do not necessarily represent those of their affiliated organizations, or those of the publisher, the editors and the reviewers. Any product that may be evaluated in this article, or claim that may be made by its manufacturer, is not guaranteed or endorsed by the publisher.
